# Solvent-Induced Lignin Conformation Changes Affect Synthesis and Antibacterial Performance of Silver Nanoparticle

**DOI:** 10.3390/nano14110957

**Published:** 2024-05-30

**Authors:** Dan Li, Liheng Chen

**Affiliations:** 1 Guangdong Provincial Laboratory of Chemistry and Fine Chemical Engineering Jieyang Center, Jieyang 515200, China; 2112106002@mail2.gdut.edu.cn; 2 Guangdong Provincial Key Laboratory of Plant Resources Biorefinery, School of Chemical Engineering and Light Industry, Guangdong University of Technology, Guangzhou 510006, China; 3 Guangdong Basic Research Center of Excellence for Ecological Security, Green Development in Guangdong-Hong Kong-Marco Greater Bay Area (GBA), Guangdong University of Technology, Guangzhou 510006, China

**Keywords:** lignin, silver nanoparticles, lignin conformation, antibiotics

## Abstract

The emergence of antibiotic-resistant bacteria necessitates the development of novel, sustainable, and biocompatible antibacterial agents. This study addresses cytotoxicity and environmental concerns associated with traditional silver nanoparticles (AgNPs) by exploring lignin, a readily available and renewable biopolymer, as a platform for AgNPs. We present a novel one-pot synthesis method for lignin-based AgNPs (AgNPs@AL) nanocomposites, achieving rapid synthesis within 5 min. This method utilizes various organic solvents, demonstrating remarkable adaptability to a wide range of lignin-dissolving systems. Characterization reveals uniform AgNP size distribution and morphology influenced by the chosen solvent. This adaptability suggests the potential for incorporating lignin-loaded antibacterial drugs alongside AgNPs, enabling combined therapy in a single nanocomposite. Antibacterial assays demonstrate exceptional efficacy against both Gram-negative and Gram-positive bacteria, with gamma-valerolactone (GVL)-assisted synthesized AgNPs exhibiting the most potent effect. Mechanistic studies suggest a combination of factors contributes to the antibacterial activity, including direct membrane damage caused by AgNPs and sustained silver ion release, ultimately leading to bacterial cell death. This work presents a straightforward, adaptable, and rapid approach for synthesizing biocompatible AgNPs@AL nanocomposites with outstanding antibacterial activity. These findings offer a promising and sustainable alternative to traditional antibiotics, contributing to the fight against antibiotic resistance while minimizing environmental impact.

## 1. Introduction

Bacterial infections represent a formidable challenge to public health globally, contributing significantly to morbidity and mortality rates [1,2]. The efficacy of antibiotics, the cornerstone of antibacterial therapy, is increasingly being compromised by the emergence of antibiotic-resistant bacteria [3,4]. This alarming trend, coupled with the prolonged and costly process of developing new antibiotics, underscores the urgent need for alternative antimicrobial strategies [5]. In this context, silver nanoparticles (AgNPs) have emerged as promising candidates due to their potent bactericidal properties and broad-spectrum activity against various bacterial strains [6,7,8,9,10]. However, despite their effectiveness, concerns regarding the toxicity of AgNPs to human cells and their potential environmental impact have raised apprehensions about their widespread use [11,12]. The interaction of silver nanoparticles with cell membranes and their propensity to release silver ions have been implicated in their cytotoxicity [13]. Moreover, the oxidation of AgNPs during storage can lead to the release of silver ions, which may have adverse effects on aquatic organisms and bioaccumulate in the food chain, posing environmental risks [14,15].

To address these challenges, there is a pressing need to develop alternative antibacterial agents that are not only effective but also safe for human use and environmentally sustainable [16]. The use of plant extracts for the synthesis of AgNPs has emerged as a green alternative approach [17]. One promising avenue for exploration is the utilization of lignin, a complex biopolymer abundant in plant cell walls, for the synthesis of antibacterial materials [18]. Lignin is the second most abundant biopolymer on Earth after cellulose [19] and represents a vast and underutilized resource with immense potential for various applications [20]. It possesses several unique properties that make it well-suited for biomedical and environmental applications [21]. Firstly, lignin exhibits non-toxicity to human cells, making it a safe and biocompatible material for medical applications [22]. Secondly, lignin possesses antioxidant properties [23], which can be beneficial for combating oxidative stress and inflammation in biological systems. Moreover, lignin has a large surface area and abundant functional groups [19,24], such as phenolic hydroxyl and methoxy groups, which can facilitate its functionalization and nanoparticle synthesis.

In recent years, there has been growing interest in the synthesis of silver nanoparticles using lignin as a reducing agent and stabilizer [25,26]. Lignin-based silver nanoparticles offer several advantages over traditional AgNPs, including enhanced biocompatibility [27], reduced cytotoxicity [28], and improved stability [29]. Furthermore, the functional groups present in lignin can impart additional properties to the nanoparticles, such as improved dispersibility [30] and targeted delivery [31]. Despite the promise of lignin-based synthesis of silver nanoparticles, several challenges remain to be addressed. One major hurdle is the water-insolubility of most lignin types [32], which necessitates the use of specific solvents for nanoparticle synthesis. Traditional methods often rely on strong polar solvents like dimethyl sulfoxide (DMSO) for AgNP formation, limiting the applicability of lignin-based synthesis in diverse solvent environments [33]. This constraint poses significant obstacles to achieving synergistic antibacterial effects with different antimicrobial agents, as specific solvents may be required to accommodate the polarity requirements of each agent.

In response to these challenges, our research team has embarked on an innovative approach to synthesize silver nanoparticles using lignin as a reducing agent and stabilizer across a range of solvent polarities. In our system, it has been observed that effective synthesis of antibacterial agents incorporating lignin-loaded silver nanoparticles can be achieved across a spectrum of solvents ranging from weakly polar to strongly polar. Furthermore, we have discovered that antibacterial agents synthesized from lignin solutions of varying polarities exhibit distinct morphologies and antibacterial activities. By exploring solvents of varying polarities, we aim to optimize the synthesis process and tailor the properties of lignin-loaded AgNPs to specific applications. This novel methodology holds the potential to revolutionize the field of antibacterial materials by providing a versatile approach to producing nanoparticles with enhanced antibacterial properties. In this paper, we present the details of our innovative synthesis approach and discuss the characterization, evaluation, and potential applications of lignin-based silver nanoparticles in antibacterial therapy. Our findings contribute to advancing the field of antibacterial materials and offer new insights into the utilization of lignin as a renewable and environmentally friendly resource for biomedical and environmental applications.

## 2. Materials and Methods

### 2.1. Materials

Alkaline lignin (AL) and enzymatic hydrolysis lignin (EHL) were procured from Shandong Longli Biotechnology Co., Ltd. (Dezhou, China), while prehydrolyzed lignin (PL) was obtained from Shandong Sun Paper Co., Ltd. (Jining, China). Silver oxide (Ag_2_O, CAS No.: 20667-12-3) with a particle size of 1 um was sourced from Aladdin Industrial Corporation (Shanghai, China), whereas MH broth and PBS buffer were acquired from Guangzhou T&R Biotech Co., Ltd. (Guangzhou, China). The glutaraldehyde solution and citric acid (CA) were purchased from Shanghai Macklin Biochemical Co., Ltd. (Shanghai, China). Gram-negative *E. coli* and Gram-positive *S. aureus* strains were sourced from Ningbo Mingzhou Biotechnology Co., Ltd. (Ningbo, China), while *P. aeruginosa* and *P. putida* strains were obtained from Guangzhou Women and Children’s Medical Center (Guangzhou, China). Artificial lysosome fluid (ALF) was prepared following established procedures outlined in the literature. Methanol, ethanol, acetone, γ-valerolactone (GVL), tetrahydrofuran (THF), dimethyl sulfoxide (DMSO), and dimethylformamide (DMF) were all analytical grade and sourced from Aladdin Industrial Corporation (Shanghai, China). All chemicals utilized in this study were of analytical grade quality.

### 2.2. One-Pot Synthesis of Silver Nanoparticles (AgNPs) Stabilized by Lignin (AgNPs@AL)

**Scheme 1:**
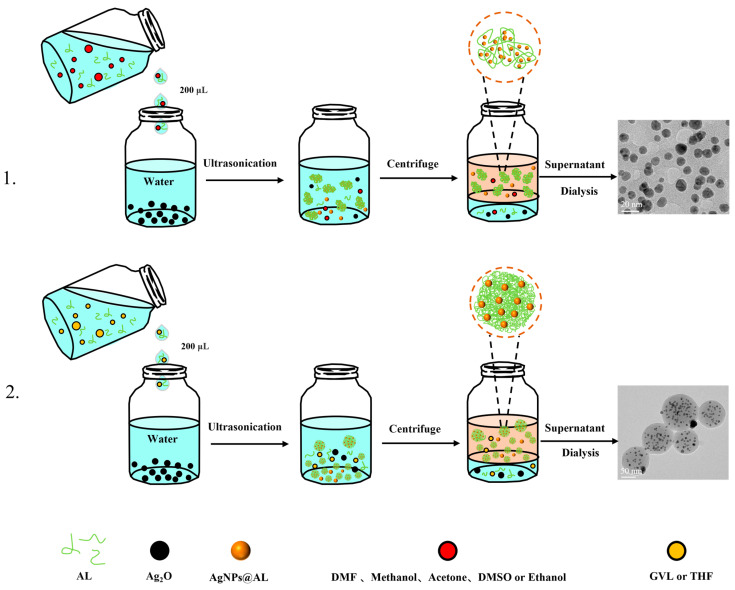
Synthesis process of AgNPs@AL from lignin solutions prepared using different solvents.

Scheme 1 illustrates the synthesis process of AgNPs@AL. In a specific experiment, 40 milligrams of alkaline lignin (AL) were dissolved in 1 mL of a binary solvent consisting of organic solvent and water in a 4:1 (*v*/*v*) ratio. The organic solvents utilized included dimethylformamide (DMF), methanol, dimethyl sulfoxide (DMSO), ethanol, acetone, tetrahydrofuran (THF), and γ-valerolactone (GVL), respectively. Subsequently, 0.2 mL of the AL solution was added dropwise to 1 mg/mL of silver oxide (Ag_2_O) aqueous suspension while subjected to ultrasonic irradiation at 360 W for 5 min (Product Model: Biosafer 900-92). Following the reaction, unreacted components were separated by centrifuging at 2000× *g* for 10 min to remove the excessive Ag_2_O. The supernatant was then dialyzed to obtain purified AgNPs@AL. In Scheme 1, the first row represents the synthesis process of nanosilver prepared using lignin dissolved by organic solvent systems of DMF, methanol, DMSO, ethanol, and acetone, respectively. On the other hand, the second row in the same figure indicates the synthesis process of nanosilver prepared using lignin dissolved by organic solvent systems of GVL or THF.

### 2.3. Characterization of AgNPs@AL

The hydrodynamic size and zeta potential of AgNPs@AL were assessed using a Zetasizer Nano ZS particle analyzer (Malvern Instruments, Malvern, UK). All reported measurements represent the average of three independent experiments. The morphology of AgNPs@AL was examined via transmission electron microscopy (TEM) using a FEI Talos F200S instrument (Hillsboro, OR, USA). The surface plasmon resonance (SPR) peak of AgNPs@AL under various conditions was analyzed employing a Shimadzu UV-2600i UV-visible spectrophotometer (Kyoto, Japan). The valence state of silver within the nanoparticles was determined using a Thermo Scientific K-Alpha+ instrument (Waltham, MA, USA) equipped with X-ray photoelectron spectroscopy (XPS). Additionally, the silver content in the nanoparticles was quantified utilizing an Agilent 720 ES instrument (Santa Clara, CA, USA) coupled with inductively coupled plasma optical emission spectrometry (ICP-OES).

### 2.4. Dissolving Behavior of AgNPs@AL

To investigate the dissolution of AgNPs@AL, samples were prepared as follows: AgNPs@AL solutions were diluted in various biologically relevant environments, maintaining a volume ratio of 1-part AgNPs@AL dispersion to 9-part biological media. These environments comprised phosphate-buffered saline (PBS, pH 7.3), artificial lysosome fluid (ALF, pH 4.1), 0.1 M citric acid solution (CA, pH 2.0), and Milli-Q water (pH 6.6) as a control. Subsequently, the mixtures were incubated at room temperature for 11 h. Absorbance measurements at the surface plasmon resonance (SPR) of AgNPs@AL were recorded hourly and normalized against the value at the initial set time.

### 2.5. Antibacterial Activity Test of AgNPs@AL Aqueous Dispersion

The antibacterial efficacy of AgNPs@AL aqueous dispersion was evaluated against a spectrum of bacteria, including Gram-negative *Escherichia coli* (*E. coli*), *Pseudomonas aeruginosa* (*P. aeruginosa*), *Pseudomonas putida* (*P. putida*), and drug-resistant Gram-positive *Staphylococcus aureus* (*S. aureus*). Following incubation of AgNPs@AL and a bacterial solution containing 10^6^ colony-forming units per milliliter (CFU/mL) in a 37 °C incubator for 1 h, 100 µL of the bacterial suspension was spread onto nutrient agar plates and then incubated at 37 °C for 24 h, after which the number of colonies was enumerated. Additionally, the effects of varying concentrations of silver nanoparticles prepared using γ-valerolactone (GVL) solvent on bacterial killing were investigated. The bactericidal ratio, determined by the number of colonies on the agar plate, was calculated as follows:

$$\mathrm{Bactericidal}\;\mathrm{ratio}\;(\%)=\frac{\left(a-b\right)}{b}\times 100\%$$

where “a” represents the number of viable colonies in the control group, and “b” denotes the number of viable colonies in the sample group.

### 2.6. Interaction between AgNPs@AL and Bacteria Observed by TEM and SEM

The samples were initially fixed in 2.5% glutaraldehyde solution (pH = 7.4) for 2 h and subsequently embedded in low melting point agarose. Following three washes with 0.1 M PBS (pH 7.2), the samples were further fixed in 1% osmium acid at 4 °C for 2 h and then subjected to dehydration using ethanol gradients. Subsequently, the samples were embedded in Epon-Araldite resin for permeabilization and placed in a mold for polymerization. Ultrathin sections were obtained for microstructural analysis, and counterstaining was performed using 3% uranyl acetate and 2.7% lead citrate [4]. The bacterial ultrastructure was visualized using transmission electron microscopy (TEM).

For scanning electron microscopy (SEM) imaging, AgNPs@AL and the bacterial mixture underwent centrifugation at 4500 rpm for 5 min. The resulting precipitate was fixed overnight with 2.5% glutaraldehyde at 4 °C. Subsequently, the fixed samples were rinsed with a PBS solution and subjected to elution with 25%, 50%, 75%, 90%, and 100% ethanol solutions for 10 min each.

### 2.7. In Vitro Cytotoxicity Assay of AgNPs@AL

The cell viability of AgNPs@AL on human embryonic kidney cells (293T) was assessed using the CCK-8 method and compared with data from previous studies on AgNPs [33]. Specifically, a suspension of 293T cells (2 × 10^4^ cells/well) was seeded onto a 96-well plate and cultured overnight in a medium containing 10% serum to facilitate cell adhesion. Following overnight incubation, samples of varying concentrations were added to the wells and incubated for 48 h. Subsequently, CCK-8 solution was added to each well, and the absorbance at 450 nm was measured using an enzyme-labeled instrument.

## 3. Results and Discussion

### 3.1. Effect of Solvents Used for Dissolving Lignin on Properties of AgNPs@AL

**Figure 1:**
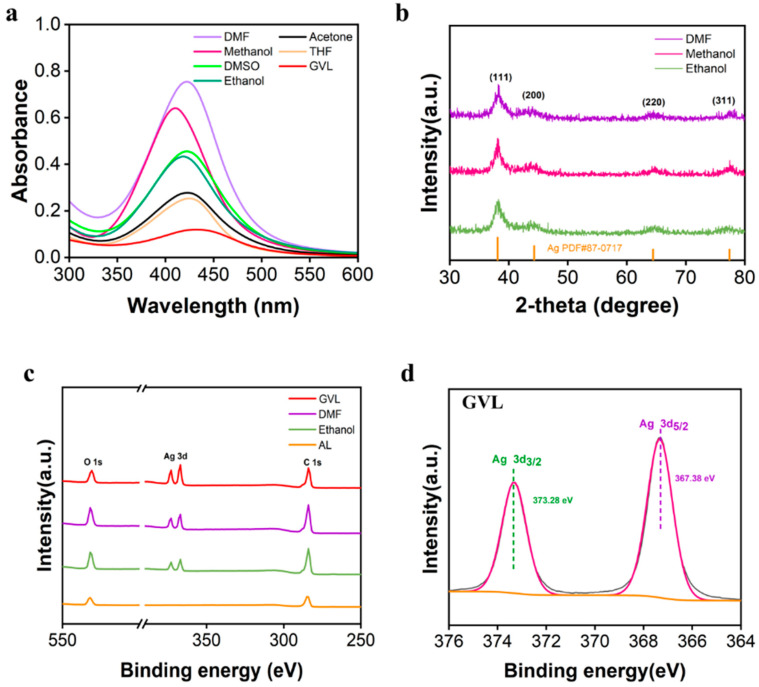
(**a**) UV-vis spectra, (**b**) X-ray diffraction (XRD) spectra, (**c**) X-ray photoelectron spectroscopy (XPS) spectra, and (**d**) high-resolution XPS survey scan of Ag 3d orbital for the synthesis of AgNPs@AL using lignin dissolved in various solvents.

The absorption spectra of all samples (Figure 1a) revealed the surface plasmon resonance (SPR) peak of AgNPs at approximately 410 nm, indicating successful AgNP formation in lignin solution using various solvents. Further characterization of the samples was conducted using XRD and XPS to confirm the composition and structure of AgNPs@AL. Figure 1b shows four peaks in the XRD pattern at 2θ values of 38.12°, 44.30°, 64.45°, and 77.40°, corresponding to the (111), (200), (220), and (311) crystallographic planes of the face-centered cubic (FCC) structure of Ag, respectively, which is in agreement with previous reports [34]. The diffraction pattern of Ag closely matched the JCPDS file No. 87-0717, with no peaks of Ag_2_O detected, indicating the high purity of the samples. Figure 1c displays the XPS spectra of AgNPs@AL synthesis from lignin solution prepared using DMF, GVL, and Ethanol, revealing Ag element peaks compared to AL, confirming successful Ag incorporation into the composites. The Ag element peak of the spectrum of AgNPs@AL produced from the GVL solvent system surpassed that from DMF and Ethanol, suggesting a higher exposure of Ag elements on the surface of AgNPs@AL from the GVL system, potentially enhancing the composite’s antibacterial activity. High-resolution XPS spectra of Ag 3d electron binding energy were deconvoluted into two peaks, as shown in Figure 1d. The characteristic binding energies of Ag 3d_5/2_ and 3d_3/2_ orbitals corresponded to those of elemental Ag, indicating that the Ag on the sample’s surface existed in the zero-valent state (Ag^0^). These results collectively confirm the successful formation of AgNPs@AL.

**Figure 2:**
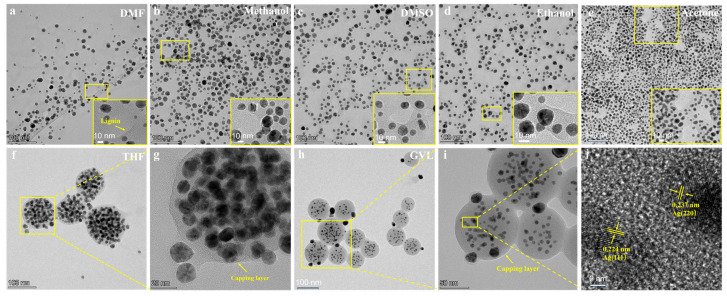
Morphologies of AgNPs@AL generated from different solvent systems are depicted. (**a**–**j**) TEM images illustrate air-dried, solvent-free suspensions after dialysis prepared from DMF, methanol, DMSO, ethanol, acetone, THF, and GVL solvent systems, while (**f**) presents AgNPs@AL crystal faces (111) and (220).

To scrutinize the morphology and structure of the samples, the dialyzed nanosilver underwent characterization via transmission electron microscopy (TEM), with results depicted in Figure 2. Due to the significantly higher electron scattering ability of AgNPs compared to lignin aggregates, when employing a bright field as the background in TEM images, AgNPs can be distinctly differentiated from lignin, where AgNPs exhibits higher contrast (i.e., darker color), while lignin appears relatively lighter (i.e., lighter color, indicated by yellow arrows in Figure 2a). Therefore, it can be seen from Figure 2a–e that Ag nanoparticles, synthesized by employing the solvents DMF, DMSO, methanol, ethanol, and acetone, were predominantly entangled within the three-dimensional network of lignin, forming sheet-like aggregates, thus stabilizing the dispersion of AgNPs. Conversely, in systems employing GVL and THF solvents, lignin-formed colloidal nanospheres (LNPs) laden with AgNPs prior to AgNPs formation, leading to the absorption of most AgNPs by the surface of LNPs, thereby forming AgNPs@AL colloidal nanoparticles [35] (Figure 2f–i). Lattice spacings observed in the TEM images (Figure 2j) were measured at 0.224 nm and 0.237 nm, consistent with the (111) and (220) plane spacings of silver, further substantiating AgNPs formation. The absorption of AgNPs by the surface of LNPs potentially facilitated the co-incorporation of other functional agents into the lignin matrix for multi-functional applications [36].

Drawing upon previous studies, we observed that the two distinct structures stemmed from the fact that GVL and THF solvents are weakly polar and capable of dissolving lignin without disrupting lignin molecule stacking [37]. Conversely, solvents with increased solvation capacity (such as methanol, ethanol, acetone, DMF, and DMSO) partially or completely dismantled the π–π stacking of lignin, impeding the formation of spherical core aggregates necessary for LNPs formation. Furthermore, regardless of solvent solvation ability, whether strong (DMF and DMSO), medium (methanol, ethanol, acetone), or weak (GVL and THF), lignin reduction of Ag^+^ to Ag^0^ followed by AgNPs formation was facilitated under ultrasonic treatment. This suggests that the reaction of lignin dissolved in various solvents with silver oxide to generate lignin-silver nanocomposites is not confined to a particular solvent but is applicable across all solvents capable of dissolving lignin.

Additionally, we employed TEM to evaluate the morphology of centrifuged nanosilver, as depicted in Figure S1, revealing consistent morphology with dialysis-derived AgNPs@AL. Moreover, scanning electron microscopy (SEM) was utilized to characterize the morphology of the centrifuged nanosilver, as shown in Figure S2, indicating the predominant existence of nanosilver on the surface of nano-lignin through chelation. The successful synthesis of AgNPs@AL was corroborated through TEM and SEM characterization. The divergent morphologies of AgNPs@AL were attributed to variances in solvent solvation abilities. The formation of AgNPs@AL heralds a novel strategy for the fabrication of lignin-based nanomaterials.

The size of AgNPs@AL in Figure 2 was then quantified using Nano Measurer 1.2 software, with the corresponding distribution histograms of AgNPs size presented in Figure S3. The effective particle size of AgNPs prepared using DMF, DMSO, methanol, ethanol, acetone, and THF was approximately 11 nm (Figure S3a–f), while the smallest AgNPs, with a size of 6 nm, were obtained using GVL (Figure S3g). It is worth noting that AgNPs@AL prepared using lignin solution dissolved by GVL showed regular spherical lignin nanoparticles (LNPs) anchoring the AgNPs. Hence, its size was also easily measured to be 87.9 ± 18.4 nm in TEM images. LNPs possess the capability to generate small AgNPs and inhibit their aggregation [35]. Furthermore, Table 1 provides a summary of the hydrodynamic diameter, polydispersity index (PDI), zeta potential, and silver content of AgNPs@AL. By adjusting different organic solvents, varied morphologies and properties of AgNPs@AL can be achieved, including AgNP content, particle size, PDI, and surface charge (Table 1). This versatility may prove advantageous for specific medical applications requiring tailored characteristics. Figures S4 and S5 demonstrated the successful synthesis of stable AgNPs@AL when our method was applied to lignin from various sources. This highlights the versatility of our methodology across different lignin sources. Simultaneously, compared to methods reported in existing literature (Table S1), our approach for synthesizing silver nanoparticles with lignin is simpler, faster, and more environmentally friendly.

### 3.2. Dissolving Behavior and Biocompatibility of AgNPs@AL

**Figure 3:**
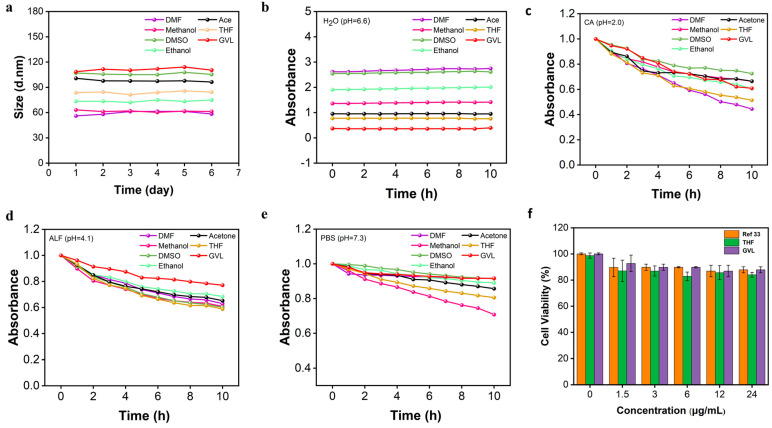
(**a**) The hydrodynamic diameter of AgNPs@AL varies with time, and AgNPs@AL dissolution trend of UV-Vis at 25 °C for 11 h in (**b**) H_2_O, (**c**) CA, (**d**) ALF and (**e**) PBS, and (**f**) the in vitro cytotoxicity of AgNPs@AL.

To assess the storage stability of AgNPs@AL, changes in particle size over time were monitored using dynamic light scattering (DLS). As depicted in Figure 3a, the particle size of AgNPs@AL remained largely unchanged over several days, suggesting favorable storage stability across samples synthesized using seven different organic solvents. Furthermore, to examine the dissolution behavior of AgNPs, AgNPs@AL was dissolved in various biological media, and alterations in the UV-Vis absorption peak of the nanosilver surface plasmon resonance (SPR) were tracked using a UV-Vis spectrophotometer according to the previous report [38]. As illustrated in Figure 3b, the characteristic absorption peak of nanosilver in H_2_O exhibited minimal change, indicating robust stability with minimal oxidation and release of silver ions. However, as demonstrated in Figure 3c, the dissolution rate of Ag^+^ was most rapid in the CA system. The dissolution rate of AgNPs in the seven samples decreased sequentially with increasing pH values. This dissolution behavior correlates with the oxidation state of nanosilver, where higher oxidation states correspond to faster dissolution rates. The strong acidity of the CA system promotes oxidation of Ag^0^ to Ag^+^, resulting in accelerated dissolution. Conversely, as pH increases, the oxidation capacity of the solution diminishes, leading to reduced dissolution rates of nanosilver (Figure 3d–f).

As analyzed from Figure 3c–e, the dissolving behaviors of AgNPs exhibited significantly different. Notably, despite theoretically expecting the fastest dissolution rate due to its smallest particle size and highest surface energy, AgNPs prepared from the GVL system exhibited the slowest dissolution rate in experimental findings. This observation may be attributed to GVL hydrolyzing to form 4-hydroxyvaleric acid (HVA) under reaction conditions [37,39]. HVA can chelate with Ag+ to form a stable complex and also reduce Ag^+^ to Ag^0^, contributing to the enhanced stability of GVL nanosilver and minimizing oxidation susceptibility. Typically, a reduction in particle size correlates with an accelerated release rate of silver ions from the nanosilver, thereby potentially increasing its toxicity [40]. Figure 3f illustrates the intriguing finding that AgNPs synthesized from the lignin-GVL solution system exhibited low cytotoxicity. Hence, despite the small size of AgNPs synthesized from the GVL solvent system, they demonstrated biocompatibility comparable to larger AgNPs [33]. This suggests that nanosilver produced under this system may exhibit both biocompatibility and excellent antibacterial properties.

### 3.3. Evaluation of Bactericidal Potential of AgNPs@AL Aqueous Dispersion

**Figure 4:**
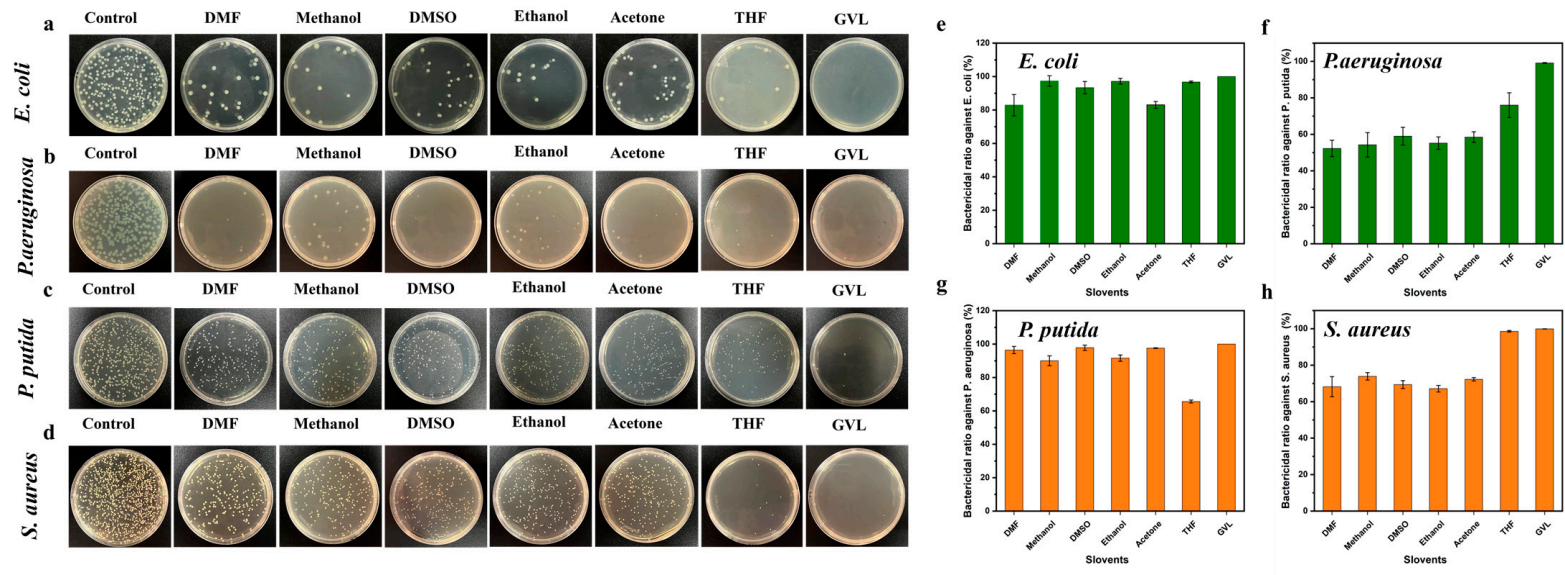
Activity determination of (**a**,**e**) *E. coli*, (**b**,**f**) *P. aeruginosa*, (**c**,**g**) *P. putidis*, and (**d**,**h**) *S. aureus* based on AgNPs@AL.

The antibacterial activities of the AgNPs@AL aqueous dispersion prepared from lignin solution with different solvents were evaluated using the plate counting method. Initially, *E. coli*, *P. aeruginosa*, *P. putida*, and *S. aureus* were exposed to AgNPs@AL at identical concentrations. To visually demonstrate the bactericidal effect, live bacterial colonies on solid nutrient agar plates exposed to different AgNPs@AL formulations for 1 h were photographed, and the bactericidal rate curve of AgNPs@AL was plotted based on colony counts. As depicted in Figure 4a, compared to the control group, the number of *E. coli* colonies in the experimental group was reduced, with no bacterial growth observed on the GVL plate, indicating effective bactericidal action of the AgNPs synthesized with seven different organic solvents against *E. coli.* Correspondingly, as shown in Figure 4e, the bactericidal rates of AgNPs@AL produced from DMF, methanol, DMSO, ethanol, acetone, GVL, and THF solvent systems against *E. coli* were 82.86%, 97.35%, 93.37%, 97.14%, 83.06%, 96.73%, and 100%, respectively, further underscoring the outstanding antibacterial efficacy of these seven nanosilver formulations against *E. coli*. Similarly, as depicted in Figure 4b–d, the number of colonies of *P. aeruginosa*, *P. putida*, and *S. aureus* in the experimental group was reduced compared to the control group. Figure 4f–h further illustrates the excellent antibacterial activity of AgNPs@AL produced from DMF, methanol, DMSO, ethanol, acetone, GVL, and THF solvent systems against *P. aeruginosa*, *P. putida*, and *S. aureus*. Additionally, it was observed that all nanosilver formulations exhibited superior antibacterial activity against Gram-positive bacteria compared to Gram-negative bacteria, likely attributable to differences in cell wall composition between the two bacterial types [25].

The antibacterial mechanism of AgNPs primarily involves several factors: release of silver ions, which interact with bacterial proteins’ thiol groups, leading to enzyme and protein inactivation and bacterial death; destruction of bacterial cell wall and membrane, causing intracellular substance leakage and bacterial death; generation of reactive oxygen species (ROS), resulting in oxidative damage to bacteria and cell death [41,42]. The antibacterial activity of AgNPs is influenced by various factors, including organic solvent type, particle size, concentration, and exposure time. In this study, nanosilver synthesized using seven different organic solvents exhibited robust antibacterial activity against all four bacteria tested. The bactericidal rate was associated with the organic solvent type and AgNPs’ particle size, with GVL demonstrating the most potent bactericidal effect, likely due to its smallest particle size [43].

These results demonstrate the effective antibacterial activity of AgNPs@AL against four bacteria and underscore the importance of organic solvent type and AgNPs’ particle size in determining antibacterial efficacy, providing insights for the future application of AgNPs@AL in antibacterial materials.

**Figure 5:**
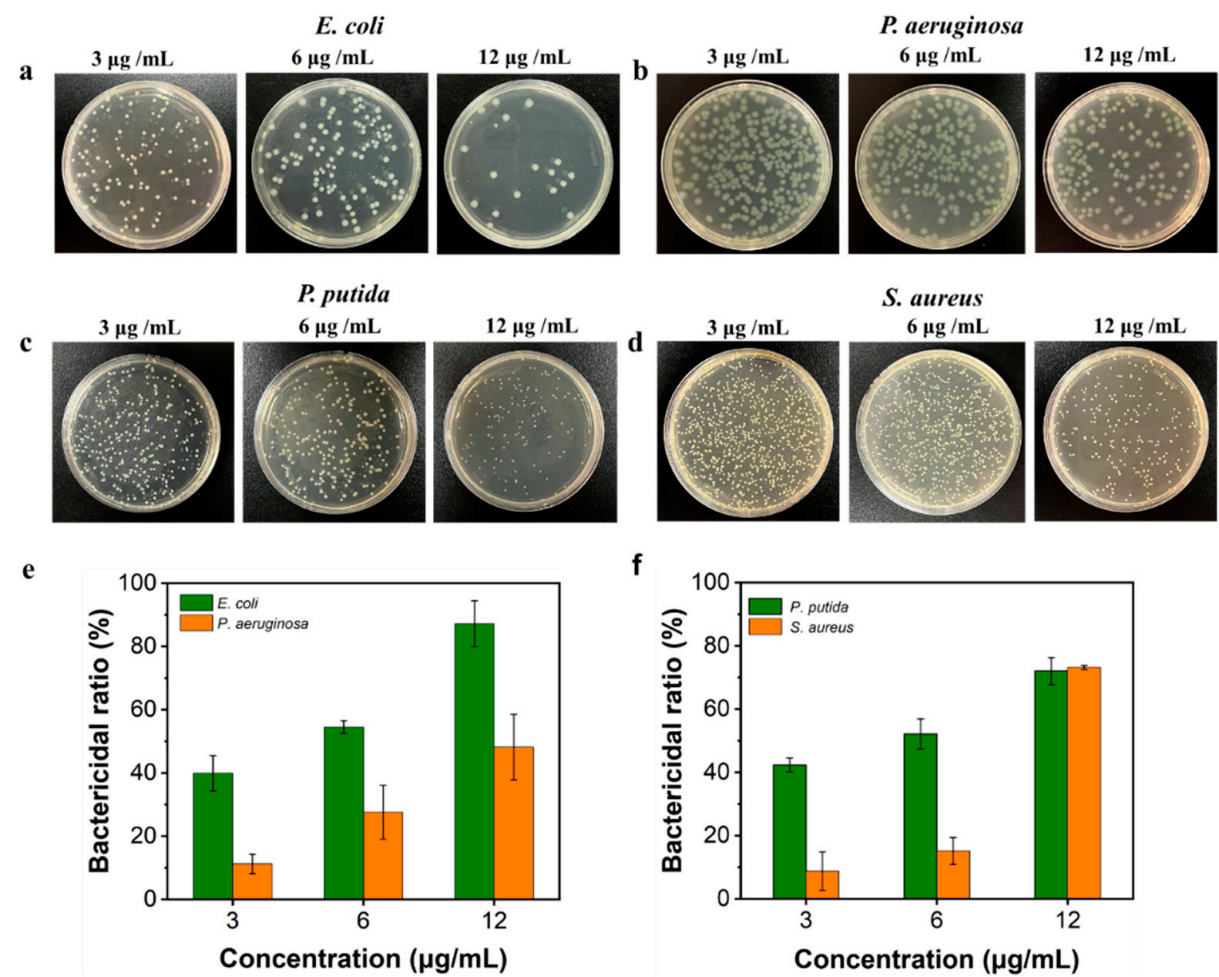
Incubate (**a**) *E. coli* (**b**) *P. aeruginosa*, (**c**) *P. putidis* (**d**) *S. aureus* for 24 h with different doses of AgNPs@AL. Activity determination of (**e**) *E. coli* and *P. aeruginosa* and (**f**) *P. putidis* and *S. aureus* at different AgNPs@AL concentrations.

We further examined the antibacterial efficacy of AgNPs@AL synthesized using GVL at varying concentrations. Initially, *E. coli*, *P. aeruginosa*, *P. putida*, and *S. aureus* were exposed to AgNPs@AL formulations prepared with GVL at different concentrations, and the number of viable colonies on agar plates was assessed. As depicted in Figure 5a–d, an increase in AgNPs@AL concentration corresponded to a decrease in the number of colonies for all four bacteria, indicating enhanced antibacterial activity with higher AgNPs@AL concentrations. Correspondingly, bactericidal rates were calculated (Figure 5e–f, demonstrating a notable dose-dependent effect of nanosilver on all four bacteria. With increasing nanosilver concentration, the bactericidal effect became more pronounced. For instance, a concentration of 12 µg/mL AgNPs@AL was sufficient to eliminate over 90% of E. coli cells, while 24 µg/mL AgNPs@AL completely eradicated all *E. coli* cells (Figure 4a GVL). Finally, based on Figure 5e,f, we calculated the IC50 (half maximal inhibitory concentration) to evaluate the antibacterial effect of AgNPs@AL [44]. The IC50 values of AgNPs@AL against *E. coli*, *P. aeruginosa*, *P. putida*, and *S. aureus* were 4.91 µg/mL, 12.19 µg/mL, 5.29 µg/mL, and 9.74 µg/mL, respectively, further demonstrating the excellent antibacterial ability of AgNPs@AL.

Previous literature reports suggested that the antibacterial potency of AgNPs escalated significantly as particle size decreased, particularly when the size fell below 10 nm [42]. Therefore, nanosilver synthesized using DMF, methanol, DMSO, ethanol, acetone, and THF solvents exhibited excellent antibacterial activity, with GVL-synthesized nanosilver demonstrating the highest efficacy. This was attributed to the increased availability of surface Ag atoms for reactive oxygen species (ROS) generation in smaller Ag crystal sizes, facilitating the release of Ag atoms and ions from the structure [45]. Consequently, damaged bacterial membranes facilitated the diffusion of Ag atoms and ions into cells, inducing damage to subcellular components and hastening bacterial cell death [46].

AgNPs can generate ROS, which induces oxidative damage to bacteria and subsequent cell death [47]. The antibacterial activity of AgNPs at different concentrations is influenced by organic solvent type, particle size, and exposure duration. In this study, AgNPs@AL prepared using GVL exhibited superior antibacterial activity against all four bacteria at varying concentrations. The bactericidal rate correlated with AgNPs@AL concentration and AgNPs’ particle size.

These findings demonstrate the effective antibacterial activity of AgNPs@AL against four bacteria at different concentrations, highlighting the relationship between antibacterial efficacy, AgNPs@AL concentration, and AgNPs’ particle size. This provides a foundation for the potential application of AgNPs@AL in antibacterial materials.

### 3.4. Antibacterial Mechanism of AgNPs@AL

**Figure 6:**
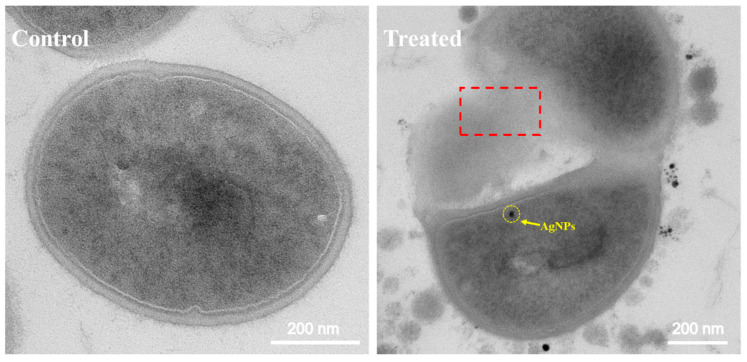
The morphology of untreated bacterial and bacterial treated by AgNPS@AL. Note: Bacterial membrane destruction is indicated by a yellow arrow.

The nanoscale dimensions of AgNPs@AL confer unique properties, facilitating interactions with bacteria distinct from larger particles. The diminutive size allows AgNPs@AL to breach bacterial cell membranes, compromising membrane integrity and inducing cell death [42,48,49]. Transmission electron microscopy (TEM) analysis was conducted to delve deeper into the antibacterial mechanism of AgNPs@AL. As depicted in Figure 6, untreated *S. aureus* cells exhibited typical morphology, featuring transparent cell walls and dense intracellular contents. Conversely, following AgNPs@AL treatment, *S. aureus* cells internalized nanosilver particles (yellow arrows), displaying compromised cell walls, ruptured membranes, content leakage, and cell death [50] (red dashed area).

**Figure 7:**
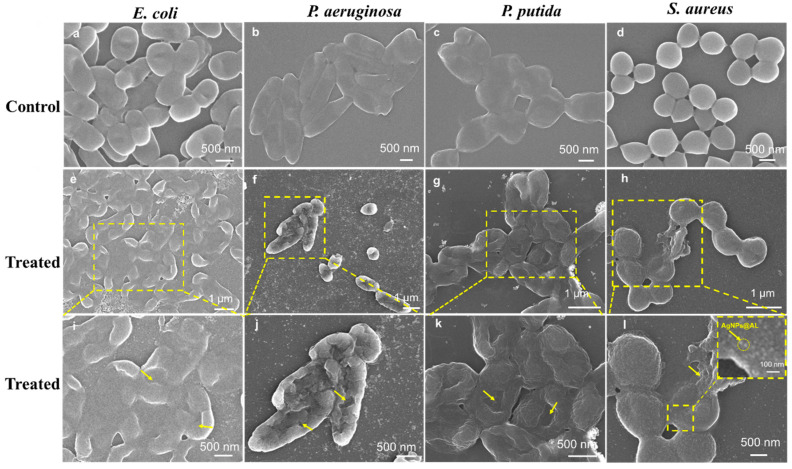
The SEM micrographs of *E*. *coli*, *P*. *aeruginosa*, *P*. *putida*, and *S*. *aureus* after incubation with PBS (control) (**a**–**d**) and AgNPs@AL (treated) (**e**–**l**) for 5 h at 37 °C. (**i**–**l**) are the magnified views of the yellow dashed regions in (**e**–**h**), respectively. Note: Bacterial membrane destruction is indicated by a yellow arrow.

To further elucidate the mechanism underlying AgNPs@AL-induced bacterial eradication, scanning electron microscopy (SEM) was employed to examine bacterial morphological changes. Untreated Gram-negative bacteria, including *E. coli*, *P. aeruginosa*, *P. putida*, and Gram-positive *S. aureus*, exhibited characteristic rod-shaped and spherical morphologies, respectively (Figure 7a–d), with smooth, intact cell wall surfaces. In contrast, AgNPs@AL-treated bacteria, both Gram-negative and Gram-positive, displayed notable morphological alterations characterized by prominent pitting or wrinkling (Figure 7e–l). These observations suggest membrane lysis and loss of cellular contents, indicative of compromised cell integrity. Energy-dispersive X-ray spectroscopy (EDX) analysis revealed substantial AgNPs@AL attachment to *S. aureus* (Figure 7i and Figure S6), suggesting a stronger affinity of Gram-positive bacteria for negatively charged AgNPs@AL compared to Gram-negative bacteria, likely due to larger contact areas [14]. This enhanced interaction likely contributes to the superior antibacterial activity of AgNPs@AL against *S. aureus*, explaining the heightened inhibitory effect on its growth across all AgNPs@AL formulations.

**Figure 8:**
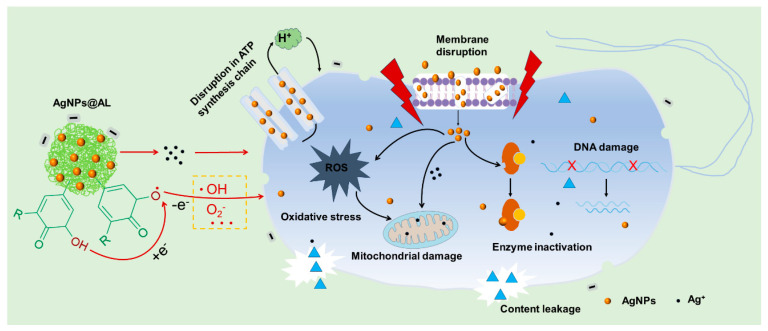
Antimicrobial Mechanism diagram of AgNPs@AL.

Drawing from prior research on the antibacterial mechanisms of lignin-based silver nanocomposites, several factors contribute to their antimicrobial activity. In addition to the established size-dependent effect, oxidative stress induced by reactive oxygen species (ROS) plays a pivotal role in bacterial inactivation. ROS generation can result from various pathways, including silver ion release and quinone/semiquinone free radical formation, subsequently yielding active oxygen species [28]. Both ROS and silver ions may enter bacterial cells, inducing oxidative stress and cell death. Therefore, we propose the following hypothetical antibacterial mechanism for AgNPs@AL, illustrated in Figure 8. The cell membrane of bacteria is directly damaged by AgNPs@AL, leading to permeability loss. Slow, sustained release of silver ions then disrupts mitochondria, inhibiting ATP production and causing DNA damage, enzyme inactivation, and ROS generation, affecting cellular components. These collective actions contribute to the antimicrobial efficacy of AgNPs@AL, ultimately resulting in bacterial demise [51].

## 4. Conclusions

This study successfully synthesized lignin-based silver nanocomposites (AgNPs@AL) using various solvents. The synthesis method is simple, adaptable, and utilizes biocompatible materials. AgNPs@AL exhibited excellent antibacterial activity against both Gram-negative and Gram-positive bacteria. The antibacterial efficacy was influenced by the type of solvent used and the size of the AgNPs. GVL-synthesized AgNPs, with the smallest size (6.2 nm), demonstrated the strongest antibacterial effect. The mechanism of action likely involves direct membrane damage, sustained silver ion release, and ROS generation within the bacteria. This research offers a promising approach for developing biocompatible and effective antibacterial materials using lignin and silver nanoparticles.

## Figures and Tables

**Table 1:** Hydrodynamic diameter, PDI, and Zeta potential of AgNPs@AL, diameter, and content of AgNPs in AgNPs@AL.

Solvents	Diameter of AgNPs@AL (nm)	PDI	Diameter of AgNPs (nm)	Zeta Potential (mV)	AgNPs Content (wt.%)
DMF	56.1	0.366	10.9 ± 2.6	−47.2	19.6
Methanol	63.2	0.264	11.7 ± 3.2	−38.3	15.2
DMSO	107.0	0.132	10.4 ± 2.7	−46.9	16.6
Ethanol	73.4	0.225	10.1 ± 3.7	−42.0	21.8
Acetone	100.7	0.198	13.5 ± 2.2	−46.6	10.9
THF	83.7	0.421	10.4 ± 2.6	−43.6	7.1
GVL	108.5	0.222	6.2 ± 2.8	−36.5	5.1

## Data Availability

Data are contained within the article and Supplementary Materials.

## Supplementary Materials

The following supporting information can be downloaded at: https://www.mdpi.com/article/10.3390/nano14110957/s1, Figure S1. Morphologies of AgNPs@AL generated from different solvent systems are depicted. (a–i) TEM images illustrate air-dried, solvent-free suspensions from DMF, methanol, DMSO, ethanol, acetone, THF, and GVL solvent systems, while (f) presents AgNPs@AL crystal faces (111) and (220). Figure S2. SEM images of DMF, methanol, DMSO, Eth, acetone, THF, GVL samples. Figure S3. (a–g) Diameter distribution of DMF, methanol, DMSO, Eth, acetone, THF, GVL samples, (h)Diameter distributions of LNP measured from TEM images, including (h) shown. Figure S4. UV-Visual spectra of AgNPs made from EHL, PL and AL respectively. Figure S5. (a–c) SEM images of AgNPs from EHL, PL and AL. Figure S6. EDAX spectrum on the surface of Staphylococcus aureus after AgNPs@AL treatment. Table S1. Comparison of synthesis methods and particle size of AgNPs@AL [7,8,21,27,28,43,52,53,54].
