# Supplementary material for: Solvent-Induced Lignin Conformation Changes Affect Synthesis and Antibacterial Performance of Silver Nanoparticle
Source: Nanomaterials (Basel). 2024 May 30;14(11):957. doi: 10.3390/nano14110957 (PMC11173806; doi:10.3390/nano14110957)
Supplement: Supplementary file 1 [file nanomaterials-14-00957-s001.zip › nanomaterials-2995319-supplementary.pdf]

Supporting Information

Solvent-induced lignin conformation changes affect synthesis and antibacterial performance of silver nanoparticle

Dan Li[a,b] and Liheng Chen[a,b,c*]

[a] Guangdong Provincial Laboratory of Chemistry and Fine Chemical Engineering Jieyang Center, Jieyang 515200, China
[b] Guangdong Provincial Key Laboratory of Plant Resources Biorefinery, School of Chemical Engineering and Light Industry, Guangdong University of Technology, Guangzhou 510006, China.
[c] Guangdong Basic Research Center of Excellence for Ecological Security and Green Development in Guangdong-Hong Kong-Marco Greater Bay Area (GBA)，Guangdong University of Technology, Guangzhou 510006, China
*Corresponding authors:
E-mail: lihengchen@gdut.edu.cn (L. Chen)

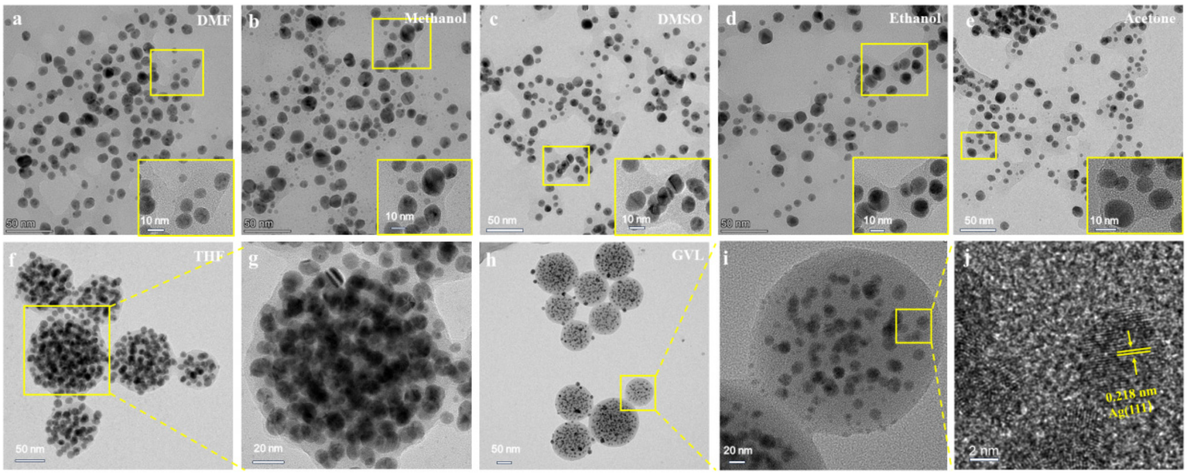

Figure S1. Morphologies of AgNPs@AL generated from different solvent systems are depicted. (a–i) TEM images illustrate air-dried, solvent-free suspensions from DMF, methanol, DMSO, ethanol, acetone, THF, and GVL solvent systems, while (f) presents AgNPs@AL crystal faces (111) and (220).

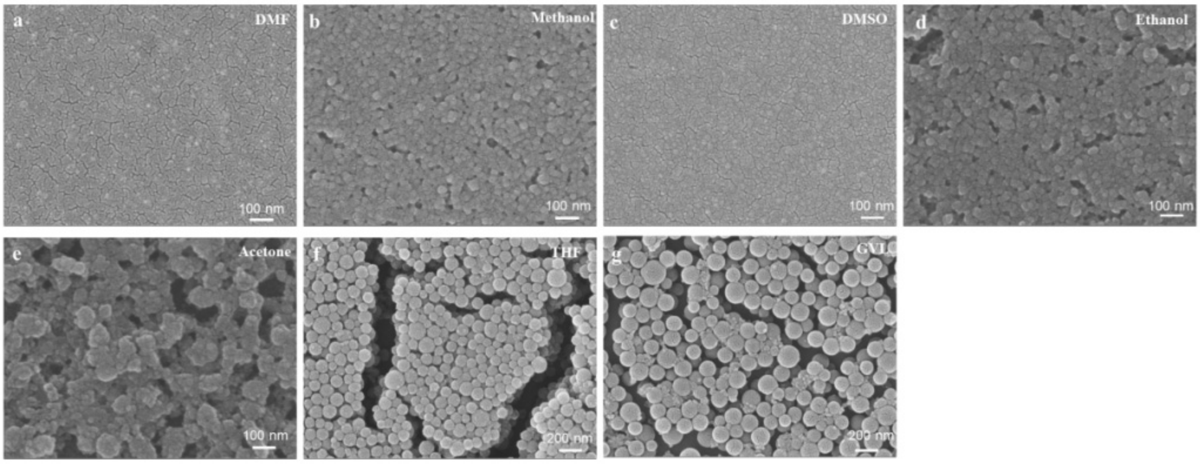

Figure S2. SEM images of DMF, methanol, DMSO,Eth, acetone, THF, GVL samples.

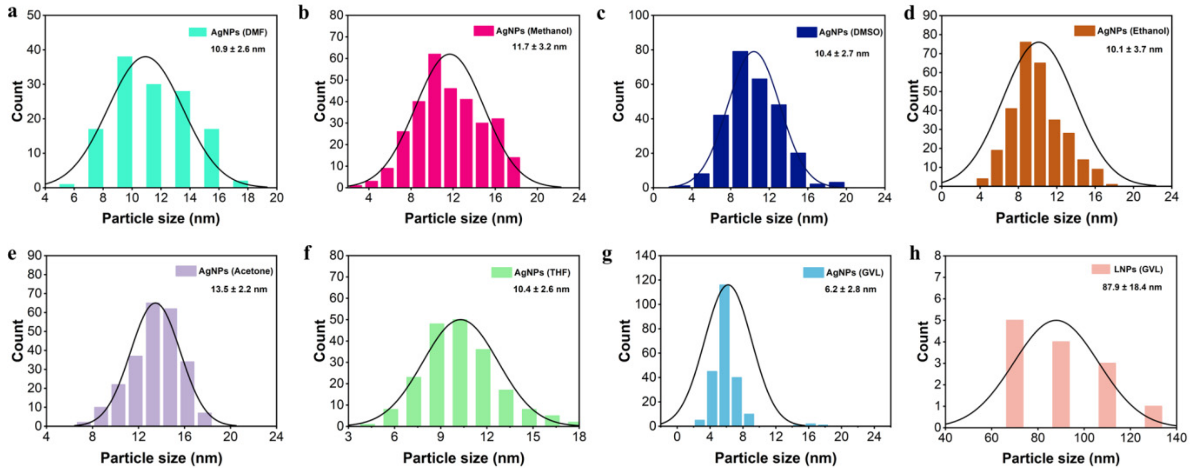

Figure S3. (a-g) Diameter distribution of DMF, methanol, DMSO,Eth, acetone, THF, GVL samples, (h)Diameter distributions of LNP measured from TEM images, including (h) shown.

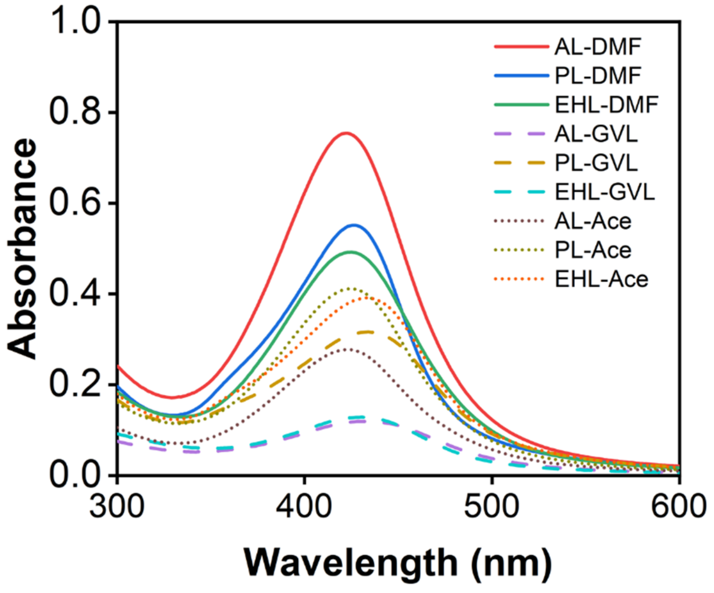

Figure S4. UV-Visual spectra of AgNPs made from EHL, PL and AL respectively.

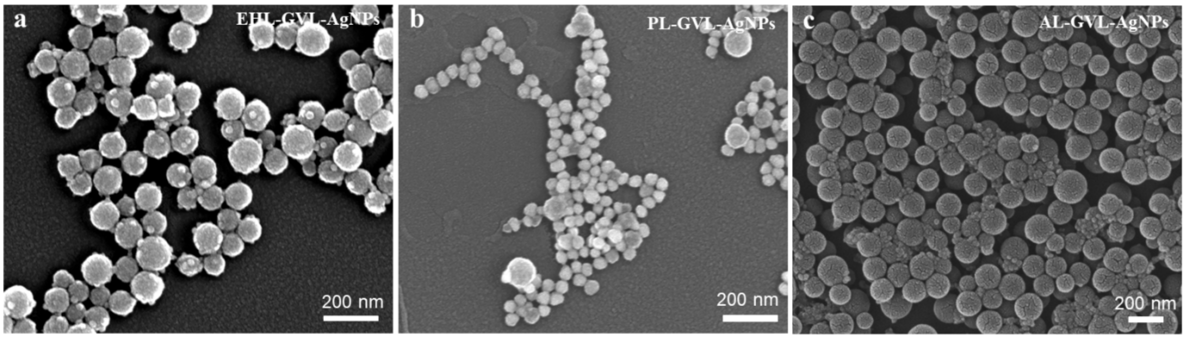

Figure S5. (a-c) SEM images of AgNPs from EHL, PL and AL.

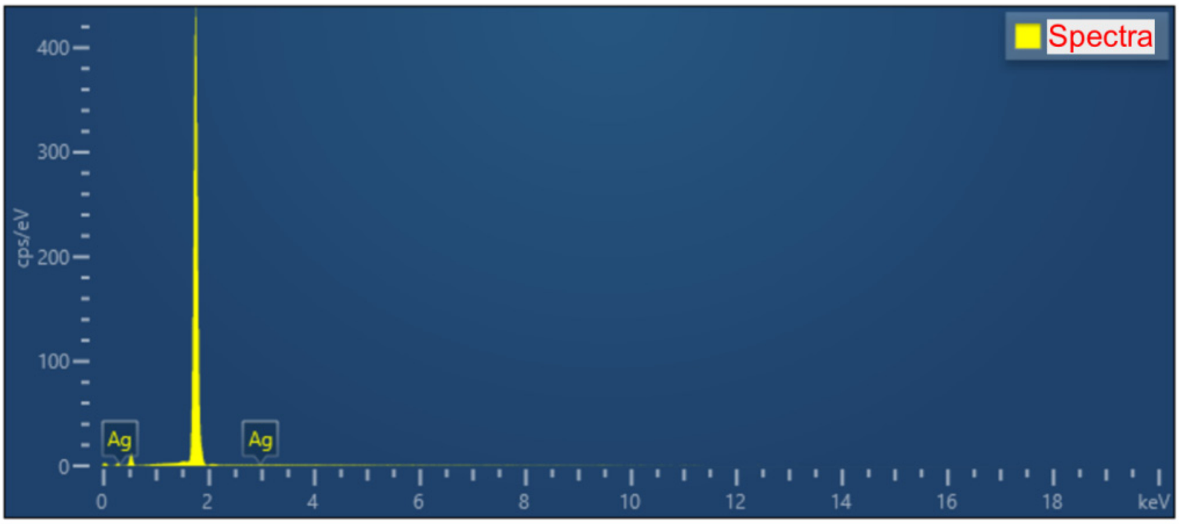

Figure S6. EDAX spectrum on the surface of Staphylococcus aureus after AgNPs@AL treatment.

Table S1. Comparison of synthesis methods and particle size of AgNPs@AL

silver precursor	reducing agent	stabilizer	condition	Purification	Size (nm)	ref
Ag_2O	alkali lignin	alkali lignin	25 °C; 12 h	centrifugation	17–27	1
$AgNO_3$	lignocellulose hydrogel	lignocellulose hydrogel	soak at 60°C for 5 h (pH = 10.0)	washing by water	9.5	2
$AgNO_3$	quaternary ammonium lignin	quaternary ammonium lignin	microwave radiation;320 W;10 min		10–20 nm	3
$AgNO_3$	lignin (low base sulfonate)	lignin (low base sulfonate)	60 °C; 3 days		~20 nm	4
$AgNO_3$	lignin (from rice husks)		100°C；6 h	filtration	8.0 ± 2.0	5
$AgNO_3$	alkaline lignin	alkaline lignin	stir	dialysis	53.2	6

$Ag(NH_3)_2OH$	lignin treated with laccase	LNP	heat: 39 °C for 4 h	centrifugation	14.7±6.6	7
$AgNO_3$	lignosulfonate and TBAB	CTAB	25 °C; 2 h	centrifugation	9.5-11.0	8
$AgNO_3$	carboxymethyl lignin		ultrasoni-cation;360 W，40 min		3.8±0.1	9
Ag_2O	alkali lignin	alkali lignin	ultrasoni-cation;360w,5min		6.2±2.8	this work

1. Mukheja, Y.; Kaur, J.; Pathania, K.; Sah, S. P.; Salunke, D. B.; Sangamwar, A. T.; Pawar, S. V., Recent advances in pharmaceutical and biotechnological applications of lignin-based materials. *International Journal of Biological Macromolecules* **2023,** *241*, 124601.
2. Zhang, L.; Lu, H.; Chu, J.; Ma, J.; Fan, Y.; Wang, Z.; Ni, Y., Lignin-Directed Control of Silver Nanoparticles with Tunable Size in Porous Lignocellulose Hydrogels and Their Application in Catalytic Reduction. *ACS Sustainable Chemistry & Engineering* **2020,** *8* (33), 12655-12663.
3. Wang, Y.; Li, Z.; Yang, D.; Qiu, X.; Xie, Y.; Zhang, X., Microwave-mediated fabrication of silver nanoparticles incorporated lignin-based composites with enhanced antibacterial activity via electrostatic capture effect. *Journal of Colloid and Interface Science* **2021,** *583*, 80-88.
4. Slavin, Y. N.; Ivanova, K.; Hoyo, J.; Perelshtein, I.; Owen, G.; Haegert, A.; Lin, Y.-Y.; LeBihan, S.; Gedanken, A.; Häfeli, U. O.; Tzanov, T.; Bach, H., Novel Lignin-Capped Silver Nanoparticles against Multidrug-Resistant Bacteria. *ACS Applied Materials & Interfaces* **2021,** *13* (19), 22098-22109.
5. Tran, N. T.; Nguyen, T. T. T.; Ha, D.; Nguyen, T. H.; Nguyen, N. N.; Baek, K.; Nguyen, N. T.; Tran, C. K.; Tran, T. T. V.; Le, H. V.; Nguyen, D. M.; Hoang, D., Highly Functional Materials Based on Nano-Lignin, Lignin, and Lignin/Silica Hybrid Capped Silver Nanoparticles with Antibacterial Activities. *Biomacromolecules* **2021,** *22* (12), 5327-5338.
6. Ran, F.; Li, C.; Hao, Z.; Zhang, X.; Dai, L.; Si, C.; Shen, Z.; Qiu, Z.; Wang, J., Combined bactericidal process of lignin and silver in a hybrid nanoparticle on E. coli. *Advanced Composites and Hybrid Materials* **2022,** *5* (3), 1841-1851.
7. Wang, L.; Wang, Q.; Slita, A.; Backman, O.; Gounani, Z.; Rosqvist, E.; Peltonen, J.; Willför, S.; Xu, C.; Rosenholm, J. M.; Wang, X., Digital light processing (DLP) 3D-fabricated antimicrobial hydrogel with a sustainable resin of methacrylated woody polysaccharides and hybrid silver-lignin nanospheres. *Green Chemistry* **2022,** *24* (5), 2129-2145.
8. Zhang, Y.; Hao, J.; Zhao, H.; Zhang, W.; Shi, G.; He, Y.; Zhou, S.; Qiao, X.; Pang, X., Preparation of Lignosulfonate@AgNPs Colloidal Nanocrystal Clusters through In Situ Reduction, Confined Growth, and Self-Assembly. *ACS Sustainable Chemistry & Engineering* **2023,** *11* (30), 11130-11139.
9. Jiang, W.; Zhang, Y.; Yang, D.; Qiu, X.; Li, Z., Ultrasonic-assisted synthesis of lignin-based ultrasmall silver nanoparticles for photothermal-mediated sterilization. *International Journal of Biological Macromolecules* **2024,** *262*, 129827.
